# TUSI Valve: Echocardiographic and Pathologic Correlates of a Novel Drug-Induced Valvular Disease—a Case Series

**DOI:** 10.1016/j.case.2026.01.003

**Published:** 2026-03-23

**Authors:** Juan Felipe Vasquez-Rodriguez, Gustavo Lemus-Barrios, Diego Rangel-Rivera, Valentina Garnica-Sepulveda, Yeisson Avila, Ramon Medina-Mur, Oscar M. Perez-Fernandez, Diego Holguín-Riaño, Gabriel Salazar-Castro, Karen Dueñas-Criado

**Affiliations:** a Department of Cardiology, Fundación Cardioinfantil–Instituto de Cardiologia, Bogota, Colombia; b Department of Cardiovascular Imaging, Fundación Cardioinfantil–Instituto de Cardiologia, Bogota, Colombia; c School of Medicine, Faculty of Health, Universidad Industrial de Santander, Bucaramanga, Colombia

**Keywords:** Heart valve diseases, 3,4-Methylenedioxymethamphetamine, Serotonin 5-HT2B receptor agonists, Echocardiography, TUSI

## Abstract

• TUSI ("pink cocaine") use may cause serotonin-mediated drug-induced valvular disease.
• Echocardiography shows reproducible thickening and restricted atrioventricular valve motion.
• Histology reveals myxoid degeneration without inflammatory infiltrate.
• Young patients with unexplained regurgitation require drug-use screening.
• Synthetic drug use poses emerging cardiovascular risks needing attention.

## Graphical abstract

**Figure:**
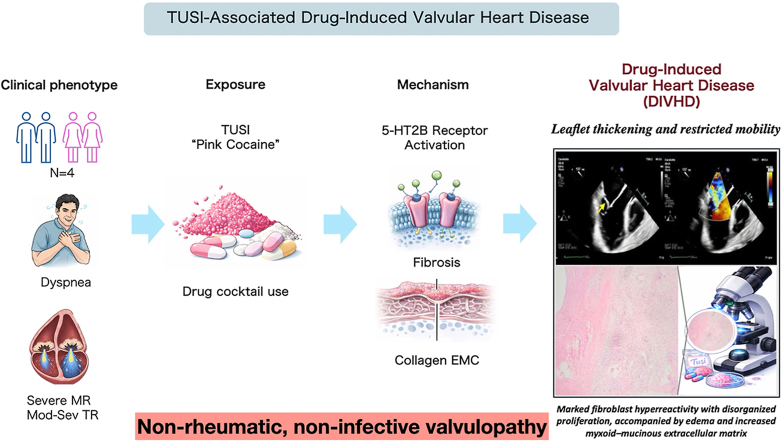

## Introduction

The global use of psychoactive, recreational, and illicit substances represents a major public health concern and continues to increase, driven by the expansion of drug markets—particularly synthetic compounds—and by widening social and economic inequalities.^1^

Along with the growing prevalence of substance abuse, several associations with cardiovascular involvement have been described, including structural valvular damage leading to mitral regurgitation (MR) and tricuspid regurgitation (TR).^2^^,^^3^

In this case series, we report the clinical, multimodality imaging, and histopathologic findings in young adults who developed significant valvular heart disease (VHD) and shared a common exposure: chronic use of a psychoactive “cocktail” known as TUSI.

## Case Presentation

Four patients—2 female patients (ages 26 and 39 years) and 2 male patients (ages 30 and 41 years)—presented at different times to the emergency department. Although their initial reasons for evaluation varied, a detailed clinical history revealed a common pattern of progressive dyspnea and functional decline over the preceding 6 months.

The 26-year-old female patient presented with palpitations and atypical chest pain. The electrocardiogram showed sinus tachycardia without ischemic changes. Physical examination revealed a hyperdynamic precordium and a grade 4/6 holosystolic murmur at the mitral area. Heart rate was 98 bpm, while blood pressure and oxygen saturation were within normal limits. Cardiac biomarkers were negative.

The 39-year-old female patient sought medical attention for fatigue and exertional dyspnea. Physical examination revealed a grade 4/6 holosystolic murmur at both the mitral and tricuspid areas, with otherwise normal vital signs.

The 30-year-old male patient presented with subacute (2-week) shortness of breath that progressed to peripheral edema and pulmonary congestion, ultimately requiring invasive ventilatory support due to respiratory failure. The physical examination was like the previous patients, showing holosystolic murmurs at the mitral and tricuspid areas, bilateral lower-extremity edema, and fine crackles in both lung fields.

Finally, the 41-year-old male patient reported 6 months of dyspnea, with new-onset fever, mild skin rash, and joint pain during the preceding week. On physical examination, there was a regular heart rate with a grade 3/6 holosystolic murmur at the mitral area. Bilateral erythema and mild tenderness were noted over the knees, along with a faint papular rash on the trunk. Laboratory tests showed normal white blood cell count, platelet count, and hemoglobin levels, with normal kidney and liver function. Three serial blood cultures—including aerobic and anaerobic media—were negative after 5 days of incubation. C-reactive protein was elevated.

Following clinical evaluation, all patients underwent transthoracic echocardiography, which demonstrated a similar pattern: severe MR along with severe TR, the latter present in all except the 41-year-old male patient, who instead exhibited mild-to-moderate TR. Table 1 depicts the quantitative and semiquantitative measurements used to classify the severity of valvular damage.

**Table 1:** Echocardiographic measurements used to classify the valve pathology

Echocardiographic parameter	26-year-old female patient	39-year-old female patient	30-year-old male patient	41-year-old male patient
Indexed LVEDV, mL/m^2^	70	64	114	75
Indexed LVESV, mL/m^2^	23	25	40	24
LVEF %	67	60	64	68
MV-VC, cm	0.68	0.65	0.70	0.84
MV-VC3D, cm^2^	0.43	0.42	0.45	0.50
MV-E wave velocity, m/s	1.8	1.7	1.7	1.6
PW-MV/PW LVOT	2.6	3.0	2.1	3.1
LVOT-VTI, cm	13	14	16	10
Tricuspid VC, cm	1.2	1.0	1.0	0.50
RVBD (mm)	54	45	60	42
Index RVBD, mm/m^2^	33	27	32	22
Tricuspid annulus, mm	45	39	44	33
Indexed tricuspid annulus, mm/m^2^	27	22	23	17

*LVEDV*, Left ventricular end-diastolic volume; *LVEF*, left ventricular ejection fraction; *LVESV*, left ventricular end-systolic volume; *LVOT-VTI*, left ventricular outflow tract velocity-time integral; *MV-E* wave velocity, peak early diastolic transmitral inflow velocity assessed by pulsed-wave Doppler; *MV-VC*, MR vena contracta width measured by two-dimensional echocardiography; *MV-VC3D*, MR vena contracta area measured by three-dimensional echocardiography; *PW-MV/PW-LVOT*, ratio of pulsed-wave Doppler velocity-time integrals obtained at the MV inflow and left ventricular outflow tract; *tricuspid VC*, TR vena contracta width measured by two-dimensional echocardiography; *RVBD*, right ventricular basal diameter. Indexed values are normalized to body surface area.

**Figure 1:**
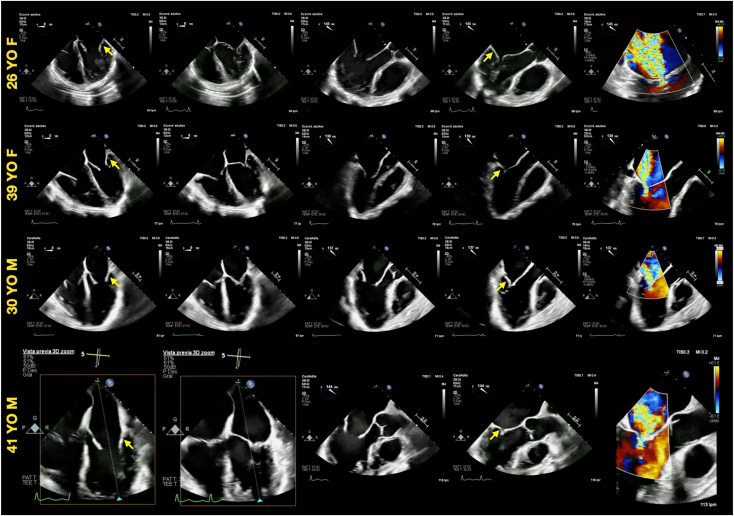
Two-dimensional morphologic assessment of the mitral valve two-dimensional TEE, midesophageal 4-chamber (0°) and long-axis (130°-145°) views, in both diastole and systole (with color-flow Doppler), demonstrates diffuse thickening of the MV leaflets—most pronounced in the posterior leaflet—and restricted leaflet closure (*yellow arrows*). All 4 patients are displayed to demonstrate the similar valve pathology with predominant involvement of the posterior leaflet and an eccentric MR jet directed inferolaterally.

**Figure 2:**
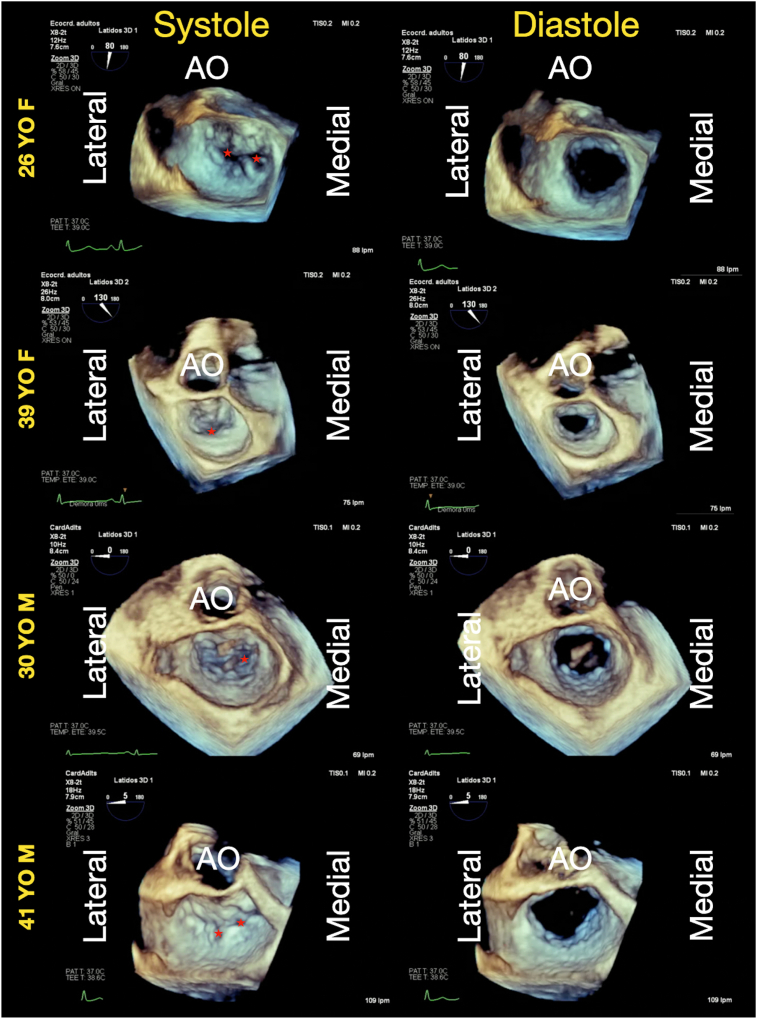
Three-dimensional morphologic assessment of the mitral valve three-dimensional TEE, volume-rendered reconstruction, surgeon's en face view displayed in systole (*left*) and diastole (*right*), demonstrates diffuse leaflet thickening with coaptation defects along the line of closure (*asterisks*) that are predominantly from restriction of the posterior leaflet during valve closure; no commissural fusion is seen in diastole. *AO*, Aortic valve.

Transesophageal echocardiography (TEE) was subsequently performed to obtain a more detailed characterization of the valvular damage. The images revealed consistent findings across all patients. The mitral valve (MV) exhibited diffuse leaflet thickening from base to tip, accompanied by restricted mobility. The limitation in leaflet motion—more pronounced in the posterior leaflet—resulted in severe eccentric MR directed posteriorly and laterally, secondary to inadequate leaflet coaptation (Figure 1, Videos 1-4). Three-dimensional reconstruction provided improved visualization of the diffuse leaflet thickening and the associated motion restriction, without evidence of commissural fusion (Figure 2, Video 5).

**Figure 3:**
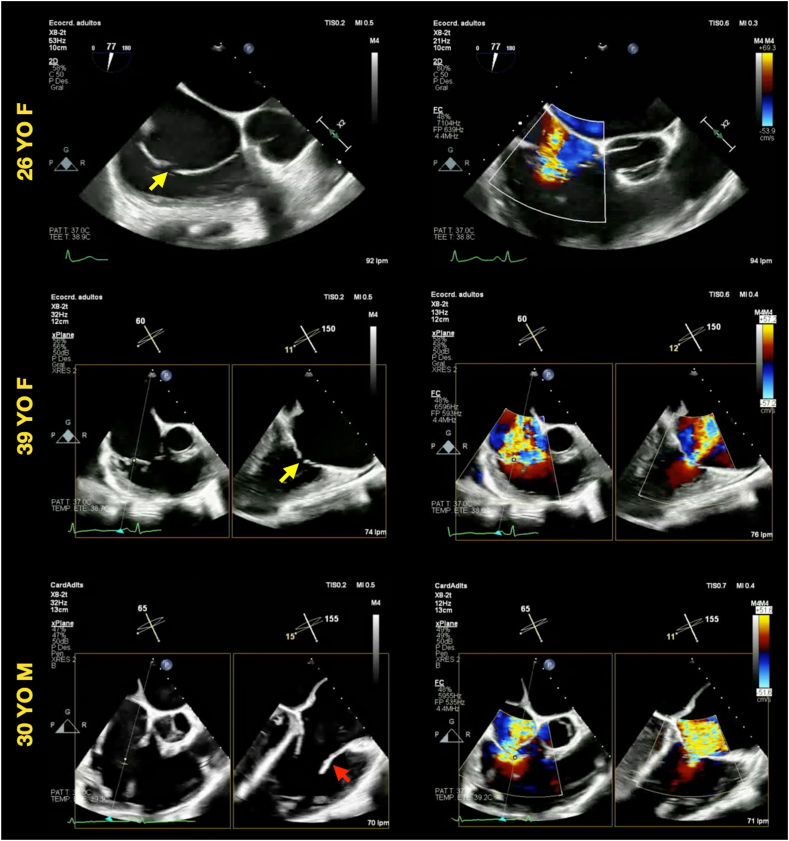
Two-dimensional morphologic assessment of the TV representative two-dimensional TEE, deep esophageal long-axis (60°-75°) view, diastolic and systolic images, without and with color-flow Doppler from 3 of the 4 patients, demonstrates the consistent findings of mild leaflet thickening (*red arrow*) and restricted systolic leaflet motion (*yellow arrows*) leading to severe TR.

The tricuspid valve (TV) was also compromised in 3 of the 4 patients (both female patients and the 30-year-old male patient). Transesophageal echocardiography revealed moderate or severe TR with morphological changes similar to those observed in the MV—namely, diffuse leaflet thickening and restricted motion (Figure 3, Videos 6 and 7). The TR was considered primary valvular regurgitation based on the abnormal leaflet morphology and normal ventricular function.

Given the morphological features described—particularly those involving the MV—and considering the setting of a developing country, rheumatic heart disease (RHD) was included as a differential diagnosis. However, the absence of commissural fusion and rheumatic stigmata, together with negative antistreptolysin O titers and rheumatoid factor in all patients, made this diagnosis less likely. Another possible explanation for the leaflet thickening was carcinoid syndrome. However, the predominant MV involvement, in the absence of a patent foramen ovale, together with the lack of symptoms suggestive of carcinoid disease, indicated a low pretest probability. In addition, urinary 5-hydroxyindoleacetic acid levels were measured in 2 of the 4 patients (both female) and were within the normal range (<6 mg/24 hour in both cases).

Due to the severity of MR, all patients underwent MV replacement. In addition, TV annuloplasty was performed in 3 of the 4 cases with concomitant severe TR. Given that all patients were younger than 45 years and had no known cardiovascular risk factors, coronary artery disease was evaluated using coronary computed tomography. No coronary lesions were identified in any of the 4 patients.

**Figure 4:**
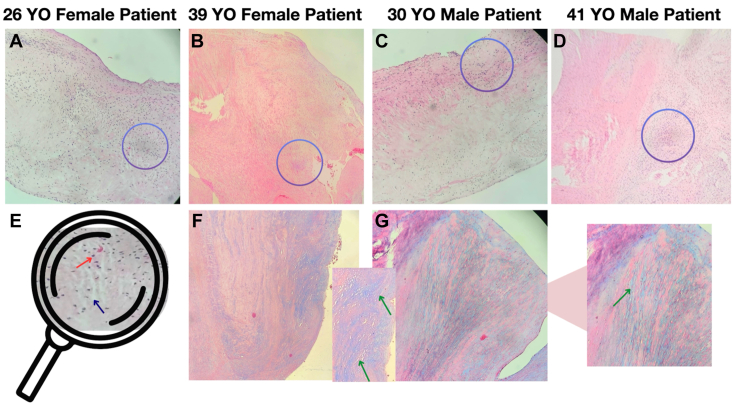
Histopathological findings from mitral valve tissue samples. Low-magnification hematoxylin and eosin (H&E, 4 × ) stains of MV tissue **(A-D)** demonstrate disorganized fibroblast proliferation, characterized by small spindle-shaped cells with punctate nuclei, associated with areas of interstitial edema and expansion of the extracellular matrix, which appear as pale, hypocellular regions. High-magnification H&E image **(E)** demonstrates an individual fibroblast (*red arrow*) and wide interstitial spaces *(blue arrow*). The focal areas circled in panels **A-D** that are more blue and suggestive of myxoid or mucinous material deposition are shown with Alcian blue staining **(F** and **G)** to demonstrate blue-stained tracts (*green arrows*) that represent increased extracellular matrix components (collagen and mucin deposition).

Histopathological analysis of the 4 patients revealed disorganized collagen architecture with expansion of the extracellular matrix, without inflammatory infiltration. Aschoff bodies were not observed. Special stains and tissue cultures were negative (Figure 4).

After comprehensive review and clinical reassessment, all patients demonstrated a similar pattern of presentation. Their functional status and the degree of MV and TV involvement were comparable, and the morphological features of both valves closely resembled one another across cases.

Further review of their medical histories revealed an additional shared factor: regular TUSI consumption (at least once per week) during the 6 months prior to presentation. The 41-year-old man also reported habitual use of 3,4-methylenedioxymethamphetamine (MDMA). Considering these exposures and the possibility of drug-related VHD, a potential causal association was proposed.

Following hospital discharge, all patients were enrolled in follow-up and cardiac rehabilitation programs. At 1-year follow-up, clinical outcomes were favorable, with no cardiovascular complications or hospital readmissions, and complete recovery of functional capacity.

## Discussion

Valvular heart disease in young individuals represents a significant clinical challenge. Although RHD remains the leading cause, a broader spectrum of etiologies has been recognized, including infectious, inflammatory, and toxin-related mechanisms.^4^ Among the latter, the potential impact of psychoactive substance use merits particular attention, especially in patients with high-risk behaviors.

The association between drug exposure and VHD was first recognized in the 1960s with ergot-derived alkaloids such as methysergide and ergotamine, and later in the 1990s with appetite suppressants like fenfluramine and dexfenfluramine, which were subsequently withdrawn from the market. Ergot-derived dopamine agonists including pergolide and cabergoline were later implicated.^2^ More recently, chronic recreational use of MDMA (“ecstasy”) has been associated with organic MR and TR.^3^

Within this expanding landscape of synthetic drugs, a new psychoactive cocktail has gained prominence in Latin America, Europe, and more recently the United States: TUSI, commonly referred to as “pink cocaine” because of its characteristic pink powder. The name derives from the phonetic pronunciation of “2C,” referencing a family of psychedelic phenethylamines, particularly 2C-B (2,5-dimethoxy-4-bromophenethylamine).^5^ In contrast to true 2C compounds, TUSI is not a single chemical entity but rather a heterogeneous mixture of psychoactive substances, most frequently containing ketamine combined with other agents, depending on local preparation. For this reason, it is broadly classified as a designer drug.^6^ Notably, despite its name, street formulations of TUSI rarely contain compounds from the 2C series, as demonstrated in multiple analytical reports.^5^^,^^7^ This substance is primarily administered via the intranasal route (sniffing).

The growing popularity of TUSI and the limited knowledge regarding its cardiovascular toxicity represent an emerging public health concern. Recent epidemiological data from nightclub-attending populations in the United States demonstrate that TUSI use was reported by approximately 2.7% of individuals, with higher prevalence among Hispanic populations. In addition, TUSI use is frequently associated with unrecognized exposure to multiple psychoactive substances, including ketamine, MDMA, and stimulants, underscoring the potential for cumulative serotonergic toxicity.^8^

In this case series, we present 4 young patients with no prior history of cardiovascular disease who developed acute or subacute VHD and shared a single common exposure: TUSI consumption. Multimodality imaging and histopathological evaluation revealed a consistent pattern of atrioventricular valve involvement, characterized by disorganized fibroblast proliferation, expansion of the interstitial extracellular matrix with mucopolysaccharide deposition, and fibrotic changes. These alterations manifested on TEE as leaflet thickening and restricted motion, with predominant involvement of the posterior MV leaflet. No alternative etiologies were identified—there was no evidence of RHD, infectious endocarditis, or primary degenerative valve pathology. Given the clinical presentation and exposure history, drug-induced VHD (DIVHD) emerged as the most plausible explanation, with the distinctive finding of its association with a relatively new recreational substance. This underscores an emerging public health concern as the use of this synthetic drug continues to increase within our community.

From an imaging perspective, the initial morphology suggested RHD; however, the absence of commissural fusion, nodular thickening, or clinical and serologic signs of streptococcal infection ruled out this diagnosis. Likewise, the thickened and retracted leaflets resembled those seen in carcinoid heart disease, although in that condition the involvement typically predominates on the right side (TV and pulmonary valve) and is associated with active neuroendocrine tumors, which were absent in our cohort. Finally, the lack of inflammatory infiltrates or Aschoff bodies on histopathology, together with the clinical course, confirmed the nonrheumatic and noninfectious nature of the process.

In our case series, we identified a shared denominator: regular “TUSI” use during the months preceding symptom onset. As previously noted, the term “TUSI” originates from the 2C series, a group of psychoactive phenethylamines known for their ability to alter perception, mood, and consciousness.^9^ However, the street mixture referred to as “TUSI” rarely contains drugs from the actual 2C series.^5^^,^^7^ The 2C compounds act as partial agonists of the serotonin receptors 5-HT2A, 5-HT2B, and 5-HT2C and are used recreationally for their psychoactive effects.^7^ In Colombia, a chromatographic analysis of multiple “TUSI” samples obtained across the country revealed that its predominant components were caffeine (96%), ketamine (96%), MDMA (88%), paracetamol (72%), and cocaine (52%). Phenethylamines from the “2C” group were detected as well, but in much lower proportions.^10^

The proposed mechanism of valvular injury is based on agonism of serotonin 5-HT_2_B receptors expressed on valvular interstitial cells (VICs).^11^ Sustained activation of these receptors promotes myofibroblast proliferation, accumulation of glycosaminoglycans, and disruption of collagen architecture, leading to progressive leaflet thickening and fibrosis.^12^ Similar mechanisms have been experimentally demonstrated with prolonged exposure to MDMA and other amphetamine analogs—compounds frequently identified in TUSI mixtures—which support the pathogenic role of 5-HT_2_B receptor activation in VICs. These studies show that G-protein-mediated receptor signaling leads to dissociation of the α and βγ subunits, followed by activation of mitogenic pathways (extracellular signal-regulated kinase/mitogen-activated protein kinase) and transforming growth factor-β signaling.^13^ These cascades promote extracellular matrix expansion, collagen accumulation, and glycosaminoglycan deposition. Droogmans *et al.*^3^ demonstrated that chronic MDMA use—one of the most frequent components identified in TUSI—is associated with a significantly higher prevalence of moderate or greater MR and TR, in a dose-dependent and cumulative manner. In vitro studies further show that MDMA, LSD, psilocin, and various 2C-series phenethylamines act as partial 5-HT_2_B receptor agonists, with binding affinities comparable to those of classic agents known to produce valvulopathy.^14^ Similar conditions have been described previously with the use of drugs such as ergotamine, methysergide, fenfluramine, pergolide, and cabergoline, among others, all sharing the same pathogenic mechanism: stimulation of 5-HT_2_B serotonin receptors on the surface of cardiac valves.^2^^,^^15^

Echocardiography plays a central role in the identification and characterization of DIVHD. Typical features include diffuse leaflet thickening extending from the base to the free edge, with initially preserved mobility followed by progressive restriction and resultant regurgitation and/or stenosis.^16^ These findings were consistently observed across all 4 patients, reinforcing the likelihood of a shared pathophysiologic mechanism.

Histopathologic analyses of DIVHD have shown that serotonin-mediated or drug-induced valvulopathy is characterized by a distinctive fibromyxoid pattern of injury, defined by diffuse leaflet thickening, expansion of the extracellular matrix, and accumulation of proteoglycans and glycosaminoglycans, accompanied by disorganized collagen architecture. These lesions typically exhibit increased numbers of VICs—particularly myofibroblasts and endothelial-derived progenitor cells—without significant inflammatory infiltration and with preservation of the valvular endothelium, distinguishing them from rheumatic or infectious etiologies.^17^ All of these features were present in the native valve tissues from our patients (Figure 4).

Our findings, together with the morphological parallels to previously described serotonergic valvulopathies, suggest a biologically plausible association between TUSI exposure and the development of DIVHD. This case series underscores how the integration of a careful substance-use history with systematic echocardiographic assessment can reveal emerging patterns of cardiovascular toxicity in young individuals. Clinicians should therefore consider serotonergic recreational drug exposure in the differential diagnosis of otherwise unexplained primary MR or TR, particularly in patients without preexisting structural or RHD.

As our knowledge advances, the development of toxicovigilance frameworks and standardized imaging protocols will be essential for early identification of drug-related valvular injury. Prospective multicenter registries integrating chromatographic analyses, receptor-binding studies, and longitudinal echocardiographic follow-up could help clarify causality and quantify the true cardiovascular risk associated with these rapidly evolving synthetic substances.

## Conclusion

The consumption of TUSI—an adulterated psychoactive mixture containing MDMA and other serotonergic analogs—may represent an emerging cause of DIVHD, mediated by activation of 5-HT_2_B receptors. The echocardiographic phenotype, histologic features, and exposure pattern observed in this case series are analogous to those described in classical serotonergic valvopathies. Targeted studies are warranted to establish causality and to define the epidemiologic impact of this underrecognized entity.

## Consent Statement

Complete written informed consent was obtained from the patient (or appropriate parent, guardian, or power of attorney) for the publication of this study and accompanying images.

## Ethics Statement

The authors declare that the work described has been carried out in accordance with The Code of Ethics of the World Medical Association (Declaration of Helsinki) for experiments involving humans

## Funding

The authors declare that this report did not receive any specific grant from funding agencies in the public, commercial, or not-for-profit sectors.

## Disclosure Statement

The authors reported no actual or potential conflicts of interest relative to this document.

## Supplementary Data

Video 1 Two-dimensional morphologic assessment of the mitral valve in a 26-year-old female patient. Two-dimensional TEE, midesophageal long-axis (145°) view, without and with color-flow Doppler, demonstrates normal left ventricular size and function, diffuse thickening of the MV leaflets with impaired coaptation, primarily restricted posterior leaflet motion, and severe eccentric MR (vena contracta 0.68 cm) with a posteriorly directed jet.

Video 2 Two-dimensional morphologic assessment of the mitral valve in a 39-year-old female patient. Two-dimensional TEE, midesophageal long-axis (130°) view, without and with color-flow Doppler, demonstrates normal left ventricular size and function, diffuse thickening of the MV leaflets with impaired coaptation, primarily restricted posterior leaflet motion, and severe eccentric MR (vena contracta 0.65 cm), with a posteriorly directed jet.

Video 3 Two-dimensional morphologic assessment of the mitral valve in a 30-year-old male patient. Two-dimensional TEE, midesophageal long-axis (137°) view, without and with color-flow Doppler, demonstrates normal left ventricular size and function, diffuse thickening of the MV leaflets with impaired coaptation, primarily restricted posterior leaflet motion, and severe eccentric MR (vena contracta 0.70 cm) with a posteriorly directed jet.

Video 4 Two-dimensional morphologic assessment of the mitral valve in a 41-year-old male patient. Two-dimensional TEE, midesophageal long-axis (144°) view, without and with color-flow Doppler, demonstrates normal left ventricular size and function, diffuse thickening of the MV leaflets with impaired coaptation, primarily restricted posterior leaflet motion, and severe eccentric MR (vena contracta 0.84 cm) with a posteriorly directed jet.

Video 5 Three-dimensional reconstruction of the mitral valve. Three-dimensional TEE, volume-rendered reconstruction, surgeon's view of the MV (left atrial perspective) in each patient case example **(A-D)**, demonstrates diffuse leaflet thickening and restricted motion with variable degrees of malcoaptation, but notably without commissural fusion.

Video 6 Representative tricuspid valve pathology in case 2 (39-year-old female patient). Two-dimensional TEE, distal esophageal long-axis (60° to 75°) simultaneous orthogonal views, displayed without **(A)** and with **(B)** color-flow Doppler, TV thickening, and restricted motion, resulting in severe primary TR; deep transgastric short-axis (52°) view, without (*left*) and with (*right*) color-flow Doppler **(C)**, demonstrates type II morphological pattern, characterized by a coaptation defect with TR origin along the line of leaflet closure; three-dimensional TEE, volume-rendered reconstruction of the TV **(D)** displayed from the perspective of the RA, demonstrates type II valvular phenotype, leaflet thickening, and restricted motion with malcoaptation.

Video 7 Representative tricuspid valve pathology in case 3 (30-year-male patient). Two-dimensional TEE, distal esophageal long-axis (65°), simultaneous orthogonal views, displayed without **(A)** and with **(B)** color-flow Doppler, demonstrates TV leaflet thickening and restricted motion, resulting in severe primary TR; deep transgastric short-axis (40°) view, without (*left*) and with (*right*) color-flow Doppler **(C)**, demonstrates type II morphological pattern, characterized by a coaptation defect with TR origin along the line of leaflet closure; three-dimensional TEE, volume-rendered reconstruction of the TV **(D)** displayed from the perspective of the RA, demonstrates type II valvular phenotype, leaflet thickening and restricted motion with malcoaptation.
